# Suicide in Chinese myths and legends—Some familiar themes

**DOI:** 10.1177/10398562231169122

**Published:** 2023-04-11

**Authors:** Saxby Pridmore, Helen J English, William Pridmore, Ahmad Naguy

**Affiliations:** 3925 University of Tasmania, Hobart, Tasmania, Australia; 5982 The University of Newcastle, Callaghan, New South Wales, Australia; 34379 Royal Hobart Hospital, Hobart, Tasmania, Australia; Kuwait Centre for Mental Health, Shuwaikh, Kuwait

**Keywords:** suicide, attempted suicide, culture, custom

## Abstract

**Aim:**

To expand our understanding of suicide by examining reports of this behavior from the Chinese mythical era (commencing circa 1200 BCE) and drawing comparisons with subsequent eras.

**Method:**

Four hundred recently published accounts of Chinese myths and folk tales were examined, along with supplementary material. Lists were created including one focused on attempted suicide and another on completed suicide. Comparisons were drawn with the suicide of a later era China and the current west.

**Results:**

No evidence was located of suicide resulting from mental disorder. Six accounts of attempted suicide and 13 of completed suicide were located. Triggers included the death of a loved one, the loss of a valued possession, complicated relationships, and the avoidance of guilt and disgrace. These accord with current western behavior.

**Conclusion:**

There is at least fair agreement in the triggers of suicide in past eras in China and the current western era. This supports the view that suicide may be, in some instances, a customary response to circumstances.

Suicide has been reported in every community in every region of the world, throughout time. During the last century the notion was pushed that suicide was always, or almost always, the result of a mental disorder.^1^ In 2014, the WHO refuted that belief allowing the topic to be studied, once again, through a wider lens.^2^ While people with mental disorder have higher suicide rates than people without mental disorder, more people live without than with mental disorder, thus suicide in the absence of mental disorder is well described.

In 1897, sociologist Emil Durkheim^3^ attributed suicide to inadequate integration of individuals into society, which left/leaves them exposed to the full impact of negative events. Culture and customs are relevant sociological concepts—culture being the set of beliefs, values, and goals shared by people living in a particular region. Those who embrace these ideas/beliefs are accepted by the community and can operate effectively in the local setting.^4^ Customs are the culturally accepted behavioral responses to particular circumstances—thus, in many instances, suicide is a culturally appropriate (customary) response.

The centrality of culture and custom to suicidal behavior is apparent in the different rates of suicide observed in different countries/regions. Consider the Philippines, a predominantly Catholic country, and South Korea, a country rejecting tradition and westernizing as rapidly as possible. Located in the Pacific Ocean, the Philippines has a suicide rate of 2.2/100 000; 2000 km directly north, on the Pacific coast, South Korea has a suicide rate of 25/100 000 [10 times greater].^5^ The differences in gender rates of suicide in different countries also illustrate the importance of culture and customs in suicide. In almost all countries, the suicide rate is greater for males than females—however, there are national differences. In China the male rate of suicide is 52% higher than the female, in Australia the male rate is 190% higher and in Oman it is 580% higher.^5^

China also clearly demonstrates the impact of culture on suicide rate. Until the 1990s, the suicide rate was higher among women than men (as in Myanmar, Bangladesh, and Pakistan). However, with cultural change in the latter part of the 20th century, female suicide fell and currently remains below the male rate.

Certain circumstances are associated with increased risk of suicide. Apprehension for crimes (especially sex-crimes against minors) carries a much higher risk of suicide. Droughts in low-income countries increase the risk for small-scale farmers. Painful and terminal illness also raises the risk.

To explore the role of social factors in suicide, we had previously studied *Romance of the Three Kingdoms*—a 14^th^ century Chinese novel.^6,7^ This work draws on oral and written history—the setting being the turbulent times of the civil war which erupted at the end of the Han dynasty (AD 180) and ended with the establishment of the Jin dynasty (AD 265). During that era, triggers for suicide included loss of status, loved ones, and fortunes—these triggers persist in current western society. ^7^

The aim of the current study was to further explore the role of culture, custom and social factors in suicide we studied the myths and legends of China. Much of this material is believed originated about 1200 BCE, being transmitted orally for more than a millennium before being recorded in writing. While precise dates are uncertain, the myths and fairy tales examined in this paper predated the material considered in our earlier study of suicide in historical China^7^ by at least 500 years.

## Method

Our primary source of information was 400 accounts of Chinese myths and folk tales collated by Barnes and Noble and published in 2020.^8^ This collection draws predominantly on five collections published in the west between 1880 and 1921. Additional material available on-line through the Project Gutenberg were also examined.^9,10^ As necessary, we placed the titles of myths/tales from Barnes and Noble^8^ in a search engine and examined the generated information.

We sought evidence of 1) mental disorder leading to suicide, 2) the view that death is/was preferable to living in unacceptable circumstances, 3) attempted suicide in response to unacceptable circumstances, and 4) completed suicide in response to unacceptable circumstances. Three tables were populated with evidence relevant to the items 2, 3 and 4 in the last sentence. Material is presented under the titles employed by Barnes and Noble^8^ and the pages from which extracts were taken are listed.

## Results

We found, 1) no accounts of mental disorder leading to suicide, 2) 13 accounts indicating individuals viewed death as preferable to their current circumstances (Table 1), 3) 6 accounts of attempted suicide in response to unacceptable circumstances (Table 2), and 4) 13 accounts of completed suicide in response to unacceptable circumstances (Table 3).

**Table 1.** Death viewed as preferable to current circumstances

Examples	Title	Page	Details
1	The Fighting Quails	34	Wang was “an idle fellow” who fell on hard times. He thought he “could not do better than cease to live.”
2	Hsiang-Ju’s Misfortunes	107	Hsiang-Ju’s wife had completed suicide. In consequence, “his sorrows made him wish to die.”
3	The Man Who Was Changed into a Crow	130	On the death of a person, another “was overwhelmed with grief, and wished to die too.”
4	The Husband Punished	192	Ching wanted to end his marriage so he could marry another person. He schemed to drive his wife away with abuse. Finally, his “wife cried out it were better she should die”
5	The Princess of the Tung-Ting Lake	217	Chen unwittingly gave offence to a princess, and confined awaiting his punishment. Hungry and anxious he concluded “death would have been almost a happy release.”
6	The Priest’s Warning	279	In Purgatory a Buddhist priest suspended upside down by a rope through a hole in his leg. “He was shrieking with pain, and longing for death.”
7	Metempsychosis	281	In Purgatory an individual resolved not to harm himself or others “but longing all the while for a happy release”
8	The Clay Image	303	A young widow being pressured by her parents to marry again stated “she would rather die”
9	Dishonesty Punished	304	A man died. This brought “great grief of his father, who would have gladly died too”
10	The King of the Snakes	455	Almond Blossom stated, “better I should die than that my father should suffer.”
11	How Greed for a Trifling Thing Led a Man to Lose a Great One	480	A man was carrying his mother when he slipped, and she was killed. “He was about to kill himself”
12	The Disowned Princess	563	Liu I stated, “I prefer to die rather than” behave dishonorably
13	The Widow and Her Son	670	A widow and her sons were starving. They had taken in an orphan girl. The mother said they must kill the girl for food. Her son said, “better kill me than the girl child”

**Table 2.** Attempted suicide in response to unacceptable circumstances

Example	Title	Page	Details
1	Miss A-pao or Perseverance Rewarded	91	A young wife - when her husband died “she hanged herself”, but was cut down. She then “refused all food.”
2	The Wonderful Stone	144	A man found a precious stone. His family sold it. In consequence, “he frequently tried to make away with himself, though luckily his servants always managed to prevent him.”
3	The Virtuous Daughter-in-Law	170	A young married woman was abused and sent back to her family of origin. Feeling disgraced she said, “I had better die” and “stabbed herself in the throat”
4	The Husband Punished	191	A young woman with family problems was found connecting a cord to a tree and stating, “there is nothing left for me but to die”
5	Metempsychosis	281	In Purgatory an individual was tortured by devils. “Thinking I had better die right off, I jumped off a cliff.”
6	The Return of Yen-Tchin-King	762	A man was threatened with being thrown into a fire. He jumped into the fire but was pulled out and saved by his captors

**Table 3.** Completed suicide in response to unacceptable circumstances

Example	Title	Page	Details
1	The Lost Brother 96	96	A young man considered by himself and others to be responsible for his brother’s death - first tried to cut his throat, later “refused all food” and died
2	Hsiang-Ju’s Misfortunes	105	A young wife was carried away by criminals. She “put an end to her existence”
3	The Quarrelsome Brothers	148	A family feud - one sister-in-law killed another with a knife and then killed herself by jumping “in a well”
4	His Father’s Ghost	256	A man was drowned. “His wife hearing the bad news swallowed poison forthwith”
5	The Boat-Girl Bride	262	A man on a boat with his new wife. He told her (as a joke) he was already married. “Before he could reach her, she was already in the river”
6	Metempsychosis	280	In a previous existence the author had been a horse and was badly treated. “At length I refused all food, and in 3 days I died.”
7	Metempsychosis	281	In purgatory a man bit his handler, “whereupon he had me destroyed”
8	Notscha	502	A man killed a dragon. Dragons captured his parents and demanded he kill himself to save them. “Very well then, I will destroy myself!”
9	Three evils	596	A man was criticized for making trouble in his community. He enlisted as a soldier and in battle placed himself in a hopeless situation to “atone for my sins” and was killed
10	How Three Heroes Came by Their Deaths Because of Two Peaches	598	Wanting rid of three soldiers a leader disgraced them. They killed themselves with their swards. “A brave hero values his honour more than his life.”
12	The Soul of the Great Bell	743	A man was instructed to cast a huge bell, but the metals would not combine. His daughter was advised by an astrologer the metals would only combine in the presence of the blood of a virgin At the next attempted casting, she said, “For thy sake, O my Father”, and jumped into the molten metal
13	The Tale of the Porcelain-God	779	A master potter, instructed by the Emperor to make a vase “with the aspect of living flesh”. After failures he “entered the flame” (of the furnace) – saying “my soul for the soul of my Vase”

## Discussion

In the myths of ancient China, we found no account of mental disorder leading to suicide. This was as expected. After years of examining historical accounts of suicide from from this era, around the globe, we have discovered only one case in which mental illness could be identified as the triggering force. This was Cleomenes I, King of Sparta, who died in 489 BCE.^11^

Mental disorder may lead to suicide—it is therefore surprising it does not appear as a trigger in the earliest oral and written accounts. However, this may not be the full story, as mental disorder was not readily recognized. Concepts of mental disorder in China were first presented in The Book of Historical documents in 1100 BCE.^12,13^ Another part of the explanation may be that when people develop severe mental disorder they function less effectively and become less prominent in society—thus, their suicides may pass relatively unnoticed. To conclude, we found no evidence of mental illness or the extant concepts of mental illness impacting on suicide rates in ancient China—that does not prove mental illness driven suicide did not occur, but it does allow that other factors may have been operative.

Thirteen examples were located of individuals stating they would prefer to die than continue living with extant circumstances. These were not individuals stating the intention to suicide—simply statements asserting, in the absence of change, death was a potential escape option. The unacceptable circumstances included the death of a loved one, pressure applied to a widow to remarry, abuse by a spouse, and prolonged physical pain.

Six examples of attempted suicide were located. All involved potentially fatal means. One person hanged herself, she was cut down, but thereafter she refused food. One person made repeated attempts, but his servants managed to prevent his death. One person jumped off a cliff (and survived the fall), another jumped into a fire but was saved by his captors. Triggering factors included the death of a loved one, the loss of a valued possession, family problems, and complicated relationships.

Thirteen examples of completed suicide were located. The means included starvation, jumping into a well, poisoning, jumping into a lake, cutting and stepping into a furnace. The motivations included the avoidance of a sense of guilt, disgrace after being raped, loss of a spouse, and the preservation of loved ones.

The circumstances described in accounts from mythical China are frequently distinct from those described in *Romance of the Three Kingdoms.* In the mythical stories reference is frequently made to supernatural entities, ghosts, fairies, elves, spirits, supernatural powers, foxes and dragons capable of transmuting into humans, and haunted dwellings. In Table 1, Examples 6 and 7 occur in Purgatory, as do Table 2 Example 5 and Table 3 Example 7. In Table 3 Example 6 is a personal account from a reincarnated man who was previously a horse, and Example 8 concerns a man captured by dragons. Example 12 concerns an artisan experiencing trouble casting an object—his daughter was advised he would only be successful if the smelting involved the blood of a virgin—consequently she jumped into molten metal. Example 13 concerns a potter seeking to make a perfect piece of porcelain—eventually he stepped into the furnace so that his soul could be incorporated into a vase.

Nevertheless, the circumstances precipitating attempted and completed suicide in the mythical era were frequently the same as those of the era of *Romance of the Three Kingdoms*, and the current era in the west. Among those who attempted suicide (Table 2) were Example 1, a person whose spouse had died; Example 2, a person who had lost a precious object; and Example 3, a person cruelly rejected by their spouse. Among those who completed suicide (Table 3) were Example 1, a person who believed himself responsible for the death of a sibling; Example 2, a woman carried away by criminals; and Example 4, a person who learned of the death of a spouse. Examples 9 and 10 involved the loss/preservation of honor.

In conclusion, we found information indicating that in ancient China, suicide has been a means of dealing with painful and difficult social situation since 1200 BC. In some instances, at least, suicide is a custom, a culturally appropriate response to circumstances—supporting evidence comes from ancient and more recent China, and from the past and present West.
